# Vacuolar Phosphatidylinositol 3,4,5-trisphosphate controls fusion through binding Vam7, and membrane microdomain assembly

**DOI:** 10.1101/2025.08.01.668199

**Published:** 2025-08-02

**Authors:** Chi Zhang, Jorge D. Calderin, Aliasgar Topiwalla, Ved Shah, Jahnavi M. Karat, Charlie T. Knapp, Razeen Ahmed, Daniel Grudzien, Elizabeth Williamson, Rutilio A. Fratti

**Affiliations:** 1. Department of Biochemistry, University of Illinois Urbana-Champaign, Urbana, IL; 2. Center for Biophysics & Quantitative Biology., Univ. of Illinois Urbana-Champaign, Urbana, IL; 3. These authors contributed equally

**Keywords:** Grp1-PH, Ypt7, SNARE, PIP3, HOPS, Vps33, PTEN, Vps34

## Abstract

Membrane trafficking is regulated by phosphoinositides (PI) and their modification by kinases, phosphatases, and phospholipases. The endolysosomal pathway is primarily controlled by PI3P, PI(4,5)P_2_ and PI(3,5)P_2_, whereas a role for PI(3,4,5)P_3_ is less clear. We report that yeast vacuoles produce PI(3,4,5)P_3_ from PI(4,5)P_2_ through class III PI 3-kinase activity. In vitro assays showed that adding dioctanoyl (C8) PI(3,4,5)P_3_ or the PI(3,4,5)P_3_-binding domain Grp1-PH blocked fusion. Furthermore, modifying endogenous PI(3,4,5)P_3_ with the phosphatase PTEN also blocked fusion. Fluorescence microscopy showed that PI(3,4,5)P_3_ was enriched at membrane vertex microdomains, which was blocked by PTEN, C8-PI(3,4,5)P_3_, and the class III PI 3-kinase inhibitor SAR405. Importantly, blocking or eliminating PI(3,4,5)P_3_ prevented the vertex enrichment of Ypt7 and the HOPS subunit Vps33. Finally, we show that the soluble SNARE Vam7 binds PI(3,4,5)P_3_ and that PTEN abolished trans-SNARE pairing between partner vesicles. Together these data indicate that vacuolar PI(3,4,5)P_3_ coordinates the assembly of microdomains and SNARE function.

## INTRODUCTION

Membrane trafficking and fusion are driven by a group of conserved regulatory proteins (e.g., SNAREs) and lipids with organelle specificity. Regulatory lipids are relatively low in abundance yet carry out critical aspects of membrane trafficking (Krauss & Haucke, 2007; Lemmon, 2008; Corvera *et al*, 1999; Balla, 2013). This group of lipids includes phosphoinositides (PI), phosphatidic acid (PA), diacylglycerol (DAG), sterols, and sphingolipids (Balla, 2013; Lingwood & Simons, 2010; Tu-Sekine *et al*, 2015; Xie *et al*, 2015; Starr & Fratti, 2019). PIs are glycerophospholipids with an inositol head group that can be differentially phosphorylated at the D-3, D-4 and D-5 positions to generate seven distinct lipids whose primary function is the binding of proteins with domains that recognize specific PIs. Broadly speaking, different organelles are marked by a dominant PI. Endosomes are marked by PI3P, and PI(3,5)P_2_, whereas the Golgi contains PI4P, and the plasma membrane is populated by PI(4,5)P_2_ and PI(3,4,5)P_3_. While concentrated at characteristic organelles, biologically significant amounts of these lipids can traffic to different membranes where they continue to signal and control membrane function.

The yeast vacuole/lysosome collects regulatory lipids from various pathways and continues to modify PIs to control homotypic vacuole fusion and vacuole fission (Starr & Fratti, 2019; Bonangelino *et al*, 2002). Homotypic vacuole fusion can be divided into at least six stages each of which is driven by a spatiotemporal-specific mixture of regulatory lipids. In a stage we now call *pre-priming*, Sec18 is sequestered from inactive cis-SNARE complexes by PA (Sasser *et al*, 2012; Starr *et al*, 2016, 2019). Sec18 can transfer to cis-SNARE complexes upon the conversion of PA to DAG by the phosphatase Pah1. Once Sec18 can engage SNAREs via its adaptor protein Sec17, *priming* occurs through ATP hydrolysis, which is dependent on ergosterol and PI(4,5)P_2_ through an undefined mechanism (Kato & Wickner, 2001; Mayer *et al*, 2000). Vacuole *tethering* occurs through the interaction of the Rab Ypt7 and its effector tethering complex HOPS (homotypic fusion and protein sorting) between partner membranes (Price *et al*, 2000b, 2000a; Seals *et al*, 2000; Zhang *et al*, 2024). Ypt7 recruitment and activation is carried out by the GEF Mon1-Ccz1 bound to PI3P (Lawrence *et al*, 2014; Cabrera *et al*, 2014), while HOPS itself can simultaneously bind several PIs including PI3P, PI4P and PI(4,5)P_2_ (Stroupe *et al*, 2006). PI3P is also essential for binding the PX domain of the soluble Qc-SNARE Vam7 and the formation of SNARE complexes during the *docking* stage (Boeddinghaus *et al*, 2002; Fratti & Wickner, 2007). Between docking and full content mixing, vacuoles can undergo hemifusion where only the outer leaflets of vesicles mix (Reese & Mayer, 2005; Reese *et al*, 2005). We and others have found that this transition requires DAG (Jun *et al*, 2004) and is sensitive exogenously added PI(3,5)P_2_ and lysophosphatidylcholine (LPC) (Miner *et al*, 2019; Reese & Mayer, 2005).

Roles for the remaining PIs (PI5P, PI(3,4)P_2_ and PI(3,4,5)P_3_) remain to be assigned in vacuole homeostasis. PI(3,4,5)P_3_ is one of the most studied phosphoinositides that is mostly present at the plasma membrane where it directs many signal transduction pathways too numerous to summarize here (Riehle *et al*, 2013). PI(3,4,5)P_3_ was discovered independently by two groups and was found to be made by class I 3-kinases p110 (α, β, δ, and γ) and the p85 regulatory domain using PI(4,5)P_2_ as a substrate (Whitman *et al*, 1988; Traynor-Kaplan *et al*, 1988; Whitman *et al*, 1985). PI(3,4,5)P_3_ is transient and its signalling is turned off by the 3’-phosphatase PTEN or the 5’-phosphatase SHIP2 (Maehama & Dixon, 1998; Pesesse *et al*, 1998). While most PI(3,4,5)P_3_ signaling is associated with the plasma membrane a significant amount is found on internal vesicles including the nuclear envelope and early endosome for localized activation of Akt (Jethwa *et al*, 2015). PI(3,4,5)P_3_ is also present on recycling endosomes for AP-1B dependent sorting (Fields *et al*, 2010), lysosomes to activate mTORC1 (Hsu *et al*, 2000), and trans-Golgi vesicles where PI(3,4,5)P_3_ on VLDL binds to the cargo receptor Sortilin/Vps10 (Sparks *et al*, 2016, 2020). At the plasma membrane itself PI(3,4,5)P_3_ recruits the protein kinases Akt and PDK1 to the membrane by binding their PH domains (Dieterle *et al*, 2014; Levina *et al*, 2022). PDK1 subsequently phosphorylates Akt to propagate signaling.

By homology, *Saccharomyces cerevisiae* lacks a class I p110 PI 3-kinase homolog, thus PI(3,4,5)P_3_ is thought not to exist in baker’s yeast. However, the fission yeast *Schizosaccharomyces pombe* also lacks a p110 homolog, yet its class III Vps34 homolog was found to make PI(3,4,5)P_3_ from PI(4,5)P_2_ in addition to its canonical product PI3P (Mitra *et al*, 2004). This suggests that a synthesis pathway for PI(3,4,5)P_3_ could have evolved prior to rise of class I PI 3-kinases. Using *S. cerevisiae* vacuoles we examined a role for PI(3,4,5)P_3_ during vacuole fusion. This study shows that PI(3,4,5)P_3_ is made on vacuoles by Vps34 and is required for efficient vacuole fusion. Removing PI(3,4,5)P_3_ with PTEN or blocking it with the Grp1-PH domain inhibited Ypt7-mediated vertex domain assembly leading to reduced trans-SNARE pairing and fusion. Finally, we found that Vam7 binds PI(3,4,5)P_3_ and PTEN leads to its release from membranes.

## RESULTS

### Short chain PI(3,4,5)P_3_ inhibits in vitro vacuole fusion.

In previous studies we have used dioctanoyl (C8) lipids to compete with endogenous sources of their long chain counterparts and various vacuolar proteins. This approach has revealed that C8-PA competes for Sec18 binding during pre-priming (Starr *et al*, 2016, 2019; Sparks *et al*, 2019), and that C8-PI(3,5)P_2_ competes for binding to the V-ATPase subunit Vph1 and regulatory factors of Ca^2+^ transport after SNARE pairing and before hemifusion (Miner *et al*, 2019, 2020; Zhang *et al*, 2022). In this study we asked whether adding C8-PI(3,4,5)_3_ affected vacuole fusion. While this lipid has not been detected in *Saccharomyces cerevisiae*, numerous screening papers have shown that baker’s yeast proteins can bind PI(3,4,5)P_3_, including the soluble SNARE Vam7 (Yu & Lemmon, 2001; Gallego *et al*, 2010; Zhu *et al*, 2001; Dunn *et al*, 2004). This could be due to lack of specificity observed in these assays, or it could indicate that the lipid may exist in small transient pools that have escaped detection.

**Figure 1.**
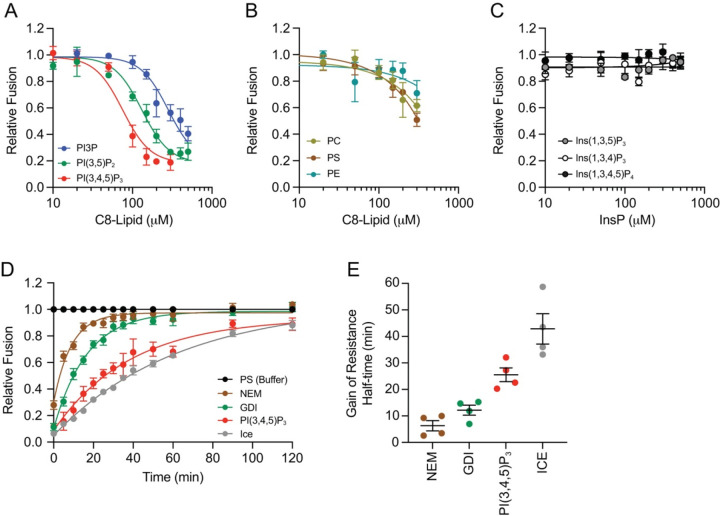
C8-PI(3,4,5)P_3_ potently inhibits fusion after vacuole tethering. Fusion reactions were treated with buffer alone or dosage curves of: C8-PI3P, C8-PI(3,5)P_2_ and C8-PI(3,4,5)P_3_ **(A);** C8-PC, C8-PS and C8-PE **(B);** Ins(1,3,5)P_3_, Ins(1,3,4)P_3_ and Ins(1,3,4,5)P_4_ **(C)** and incubated for 90 min at 27°C. Fusion was normalized to the maximum fusion (buffer alone) set to 1 for each curve. Data points show the average of multiple experiments (n≥3) and SE. Each set was fit to one-phase decay curves. IC_50_ values were determined using Graphpad Prism 10. **(D)** Gain of resistance fusion reactions were performed with buffer alone, 1 mM NEM, 2 μM GDI or 150 μM C8-PI(3,4,5)P_3_. Individual reactions were treated with reagents or buffer at the indicated time points. A second set of buffer-treated reactions were placed on ice at the indicated times. Fusion reactions were incubated for a total of 120 min. The amount of fusion for each reaction was normalized to the untreated control for the indicated time point at 27°C set to 1. Data points show the average of multiple experiments (n≥3) and SE. Each set was fit to one-phase decay curves. **(E)** Calculated half-times of resistance from assays in **(C)**. Error bars represent SEM (n=3)

Here we added exogenous C8-PI(3,4,5)P_3_ to in vitro homotypic vacuole fusion reactions and found that C8-PI(3,4,5)P_3_ potently inhibited fusion with an IC_50_ value of ~80 μM (Fig. 1A). This showed that vacuole fusion was more sensitive to C8-PI(3,4,5)_3_ compared to C8-PI(3,5)P_2_ with an IC_50_ values of ~140 μM or C8-PI3P, which failed to fully inhibit fusion at 500 μM (Miner *et al*, 2019). To confirm that the measured impacts were PI-specific and not an artifact of the C8 chains, we tested the C8 variants of PC, PE and PS and found that none of the bulk lipids had a significant effect on fusion (Fig. 1B), signifying that the C8 chains themselves had no effect and that the PI headgroups were responsible for altering vacuole fusion efficiency. While many PI binding domains such as the Plc1δ PH insert a loop into the membrane to further stabilize their interaction (Herzog *et al*, 2016; Lemmon, 2008), they can also simply bind headgroups in solution, (Kavran *et al*, 1998; Ferguson *et al*, 2000) albeit with different affinities compared to full lipids. Based on this we asked if the headgroups alone could interfere with vacuole fusion. We added the PI(3,4,5)P_3_ head group Ins(1,3,4,5)P_4_ as well as other variants (Ins(1,3,4)P_3_, Ins(1,3,5)P_3_) to fusion reactions and saw that they had no effect even when at present at 500 μM (Fig. 1C). This suggested that the head group alone was insufficient for vacuole fusion interference.

### C8-PI(3,4,5)P_3_ can inhibit in vitro vacuole fusion after docking.

In order to determine which stage of fusion was affected by C8-PI(3,4,5)P_3_ we performed temporal gain of resistance experiments (Mayer *et al*, 1996; Haas *et al*, 1995; Ungermann *et al*, 1998; Price *et al*, 2000b; Sasser *et al*, 2012; Miner *et al*, 2019). Inhibitors were added at different timepoints starting at T=0 min and at 5,10, and 30-min intervals for a total of 120 min. As reactions passed a stage of fusion, e.g. Sec18-mediated priming they became resistant to inhibitors of that stage such as antibodies against Sec18 and Sec17, NEM, propranolol, C8-PA or the small molecule IPA (inhibitor of priming activity) (Mayer *et al*, 1996; Sasser *et al*, 2012; Starr *et al*, 2016; Sparks *et al*, 2019). In this study we used NEM to mark the priming threshold, and GDI to mark the tethering/docking phase (Mayer & Wickner, 1997). Tethering and docking cannot be separated by this assay. Individual reactions were treated with buffer alone at 27 °C for the duration of the experiment, while a group of buffer-treated tubes were removed and placed on ICE to mark the maximum amount of fusion for any recorded time point. We added 150 μM C8-PI(3,4,5)P_3_ to individual reactions at the indicated times to see which stage would be impacted by lipid treatment. We found that the gain of resistance curve for C8-PI(3,4,5)P_3_ was shifted to the right of GDI indicating that it continued to affect fusion after Ypt7 mediated tethering (Fig. 1D). This was further illustrated when the half-times of resistance were calculated. Figure 1E shows that priming and docking were completed by 5 and 12 min, respectively, whereas the added C8-PI(3,4,5)P_3_ had later half-time of ~26 min. This half-time is later than the T1/2 ~15 min we saw with PI(3,5)P_2_ (Miner *et al*, 2019). Importantly, it should be noted that gain of resistance assay only indicates the last step the variable molecule had an effect; implying earlier stages could have been impacted as well.

### PI(3,4,5)P_3_-specific inhibitors block vacuole fusion.

**Figure 2.**
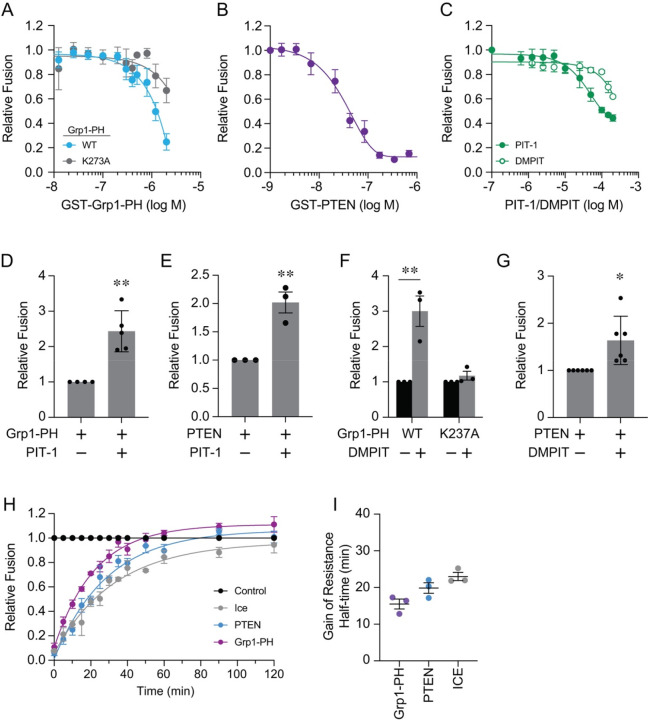
PIP3 specific reagents inhibit vacuole fusion. Fusion reactions were treated with buffer alone or dose response curves of: GST-Grp1-PH and GST-Grp1-PH^K237A^ **(A)**; GST-PTEN **(B)**; PIT-1 and 3,5-dimethyl PIT-1 (DMPIT) **(C)** and incubated for 90 min at 27°C. Each curve was normalized to the maximum fusion (no treatment) set to 1. Data points shown are the averages of multiple experiments (n≥3) and SE. Each data set was fit to one-phase decay curves and IC_50_ values were determined using Graphpad Prism 10. **(D)** Fusion inhibition by PTEN (200 nM) was rescued with 200 μM PIT-1. Fusion efficiency was normalized to PTEN alone set to 1. **(E)** Fusion inhibition by Grp1-PH (2.3 μM) was rescued with 200 μM PIT-1. Fusion efficiency was normalized to Grp1-PH alone set to 1 **(F)** Fusion inhibition by wild type Grp1-PH and Grp1-PH^K237A^ (2.3 μM) was rescued with 200 μM DMPIT. Fusion efficiency was normalized to Grp1-PH or Grp1-PH^K237A^ alone set to 1. **(G)** Fusion inhibition by PTEN (200 nM) was rescued with 200 μM DMPIT. Fusion efficiency was normalized to PTEN alone set to 1. **(H)** Gain of resistance fusion reactions were performed with buffer alone, 1 mM NEM, 2 μM GDI 2 μM GST-Grp1-PH, or 200 nM PTEN. Reactions were treated with reagents or buffer at each time point. A second set of untreated reactions was placed on ice at each timepoint. Fusion reactions were incubated for a total of 120 min and fusion for each reaction was normalized to the untreated control for the each timepoint at 27°C set to 1. Data sets show the average of multiple experiments (n≥3) and SE. Each set was fit to one-phase decay curves. **(H)** Calculated half-times of resistance from assays in **(G)**. Error bars represent mean ± SE (n=3). In panels D-G, significance was determined unpaired two-tailed t-test. * *p*<0.05, ** *p* < 0.01.

The data presented above suggests that adding C8-PI(3,4,5)P_3_ affected fusion by either disrupting membrane biophysical properties or specific protein-lipid interactions. In other words, a key PI(3,4,5)P_3_-protein interaction needed for fusion could have been disrupted by exogenous amounts of C8-PI(3,4,5)P_3_. To further test for a required PI(3,4,5)P_3_-protein interaction we used purified GST-Grp1-PH, a PI(3,4,5)P_3_-specific binding PH domain from the ARF GEF Grp1 (Guillou *et al*, 2007; Chen *et al*, 2012; Corbin *et al*, 2004; Dowler *et al*, 2000). Sequestering PI(3,4,5)P_3_ away from natural binding partners with GST-Grp1-PH potently inhibited fusion with an average IC_50_ of ~1 μM with batch-to-batch variation. This further indicated that the lipid was present on vacuolar membranes (Fig. 2A). We also tested the Grp1-PH^K273A^ mutant that has reduced PI(3,4,5)P_3_ affinity (Lindsay *et al*, 2006; Naughton *et al*, 2016; Guillou *et al*, 2007; Yamamoto *et al*, 2020). We found that it only interfered with fusion with an IC_50_ of 3.6 μM, which was non-specific as shown below.

To further probe for the presence of PI(3,4,5)P_3_ on isolated vacuoles we used the lipid phosphatase PTEN that converts PI(3,4,5)P_3_ to PI(4,5)P_2_ (Maehama & Dixon, 1998; Lee *et al*, 1999). We added a dose-response curve of purified GST-PTEN to fusion reactions and found that it inhibited with an IC_50_ of 60 nM (Fig. 2B). This further indicated that endogenous vacuolar PI(3,4,5)P_3_ affected homotypic vacuole fusion. While PTEN does have some activity against PI(3,4)P_2_ and PI3P, it is 3–5 times weaker to its activity against PI(3,4,5)P_3_ (Lee *et al*, 1999).

We then examined if we could block PI(3,4,5)P_3_ indirectly with a competitive inhibitor that targets the binding pocket of interacting proteins. We used the PITenin PIT-1, a small molecule that binds the PI(3,4,5)P_3_ binding pocket of PH domains including those of AKT, PDK1, Grp1 and ARNO (Miao *et al*, 2010). Notably PIT-1 does not interfere with PI(3,4)P_2_ interactions with TAPP1 or TAPP2 or PI(4,5)P_2_ interactions with PLC (ibid). We found that PIT-1 on its own inhibited fusion with an estimated IC_50_ of ~ 65 μM, which was near the IC_50_ for its inhibition of Akt-PH-PI(3,4,5)P_3_ interactions (ibid) (Fig. 2C). These data indicated that a native vacuole protein bound to PI(3,4,5)P_3_ to affect vacuole fusion. In addition, we used the PIT-1 derivative 3,5-dimethyl PIT (DMPIT) and observed little interference with fusion (Fig. 2C).

Next, we asked if PIT-1 could rescue the effects of Grp1-PH and PTEN. Fusion reactions were first treated with 200 μM PIT for 5 min at 27°C. Reactions were stopped by placing them on ice followed by the addition of 2 μM Grp1-PH or 200 nM PTEN. Reactions were further incubated at 27°C for a total of 90 min. This showed that PIT-1 could reverse the effects of both Grp1-PH (Fig. 2D) and PTEN (Fig. 2E). Similarly, 3,5-DMPIT rescued Grp1-PH inhibition (Fig. 2F). Interestingly, 3,5-DMPIT was not able to reverse inhibition by the Grp1-PH^K237A^ mutant further indicating that its inhibition was a result of non-specific interactions (Fig 2D, F). We also observed that 3,5-DMPIT moderately rescued the effect of PTEN (Fig. 2G).

Lastly, we tested if PTEN and Grp1 had gain of resistance curves that matched the C8-PI(3,4,5)P_3_ curve. Using the assay described above we found that the resistance curves of PTEN and Grp1-PH were shifted to the right of the GDI curve with half-times of ~25 min, which was in accord with the C8-PI(3,4,5)P_3_ data (Fig. 2H–I).

### PI(3,4,5)P_3_ was detected on vacuoles

**Figure 3.**
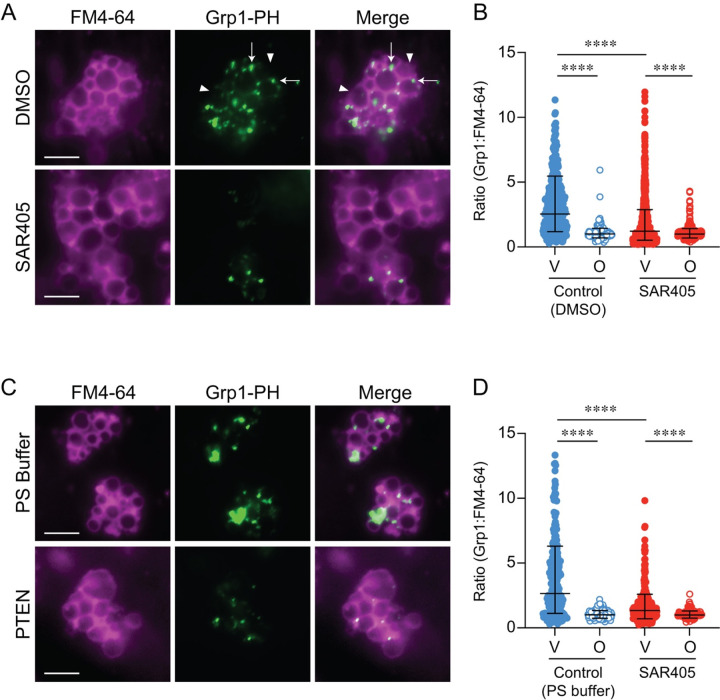
PI(3,4,5)P_3_ is made by Vps34 and localizes at the vertices of docked vacuoles. Docking reactions were incubated for 30 min at 27°C with 150 nM GST-Grp1-PH to mark PI(3,4,5)P_3_. Grp1-PH was visualized by adding CF488-anti GST polyclonal antibody (1:500) at the end of the reaction and 5 μM FM4–64 to label entire vacuoles. Vacuoles were pelleted (5,000 g, 5 min, 4°C) to remove excess antibody fluorescence and resuspended in PS buffer. Docking reactions were treated with: 200 μM SAR405 or DMSO **(A)**; 50 nM PTEN or PS buffer alone **(C)**. Reactions were mixed 1:1 with 0.6% low melt agarose and prepared for fluorescence microscopy. **(B)** Quantitation of ratiometric fluorescence intensities of vertices (V) and outer edge (O) in panel A. Data points were pooled from multiple experiments and each experiment contained ratios from 10 or more fields with 10–15 clusters made of ≥ 5 vacuoles (n > 500 vertices and > 200 outer edges for each condition). Error bars represent geometric means ± geometric SD (n=3). Significance was determined using one-way ANOVA for multiple comparisons. Tukey’s post-hoc test of multiple comparison was used for individual *p*-values. **** *p* < 0.0001. Scale bars: 5 μm.

The inhibition of fusion by PTEN and Grp1-PH suggested that PI(3,4,5)P3 was present on vacuoles. To visualize where PI(3,4,5)P3 was on docked vacuoles we used subinhibitory concentrations of GST-Grp1-PH (150 nM) and fluorescent (CF488) anti-GST antibody. This prevented any interference caused by conjugating primary amines or free surface Cys with reactive dyes. Grp1 has key Lysines in the lipid binding pocket and a Cysteine next to a lipid interacting Lys (Cronin *et al*, 2004; Lai *et al*, 2013). We found that CF488-Grp1-PH localized to vertices of docked vacuoles where essential lipids and proteins accumulate to trigger fusion (Fig. 3 A–B) (Wang *et al*, 2002; Eitzen *et al*, 2002; Wang *et al*, 2003; Fratti *et al*, 2004; Karunakaran *et al*, 2012; Jun *et al*, 2006; Miner *et al*, 2019, 2020; Zhang *et al*, 2024). Vacuole labeling was significantly reduced with the Class-III PI 3-kinase-specific inhibitor SAR405 (Ronan *et al*, 2014). This indicates that PI(3,4,5)P_3_ production could be Vps34-dependent and not due to an unidentified Class I homolog. This is also in keeping with Vps34 production of PI(3,4,5)P_3_ seen in fission yeast (Mitra *et al*, 2004). In parallel we treated vacuole with PTEN prior to adding CF488-Grp1-PH. In Figure 3C–D we show that PTEN blocked labeling by CF488-Grp1-PH showing that PI(3,4,5)P_3_ was largely eliminated from vacuoles.

### CF488-Grp1-PH labeling of vacuoles was blocked by C8-PI(3,4,5)P_3_

**Figure 4.**
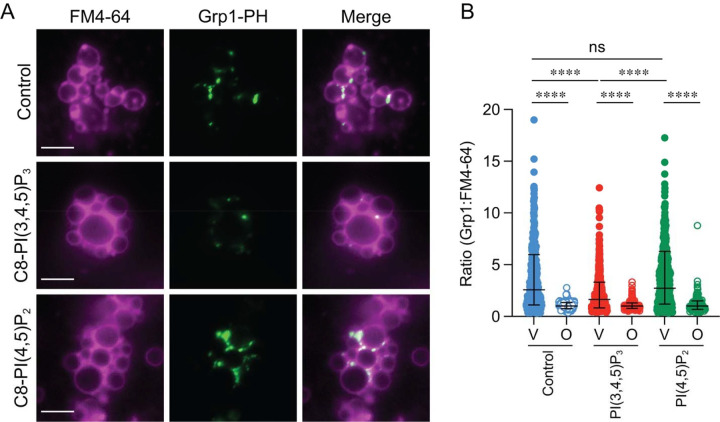
PI(3,4,5)P_3_ competes Grp1-PH binding to vertices **(A)** Docking reactions were incubated for 30 min at 27°C with 150 nM GST-Grp1-PH and visualized by adding CF488-anti GST antibody as described in Figure 3. Docking reactions were treated with 300 nM C8-PI(4,5)_2_, C8-PI(3,4,5)P_3_, or buffer alone. At the end of the incubation period, reactions were placed on ice, labeled FM4–64 and prepared for fluorescence microscopy as described. **(B)** Quantitation of ratiometric fluorescence intensities of vertices (V) and outer edge (O) in panel A. Data points were pooled from multiple experiments as described in Figure 3. Error bars represent geometric means ± geometric SD (n=3). Significance was determined using one-way ANOVA for multiple comparisons. Tukey’s post-hoc test of multiple comparison was used for individual *p*-values. **** *p* < 0.0001. NS, not significant. Scale bars: 5 μm.

Treating vacuoles with PTEN blocked CF488-Grp1-PH from accumulating on docked vacuoles through converting PI(3,4,5)P_3_ to PI(4,5)P_2_. To confirm that this was not an indirect effect we next tested CF488-Grp1-PH localization by adding subinhibitory levels (300 nM) of C8-PI(3,4,5)P_3_ or C8-PI(4,5)P_2_ as competitive inhibitors. This showed that CF488-Grp1-PH labeling was inhibited by C8-PI(3,4,5)P_3_ but not C8-PI(4,5)P_2_ (Fig. 4A–B). This further demonstrated CF488-Grp1-PH vertex enrichment was specifically due to PI(3,4,5)P_3_ binding.

### PI(3,4,5)P_3_ affected GFP-Ypt7 and HOPS enrichment at vertex domains

**Figure 6.**
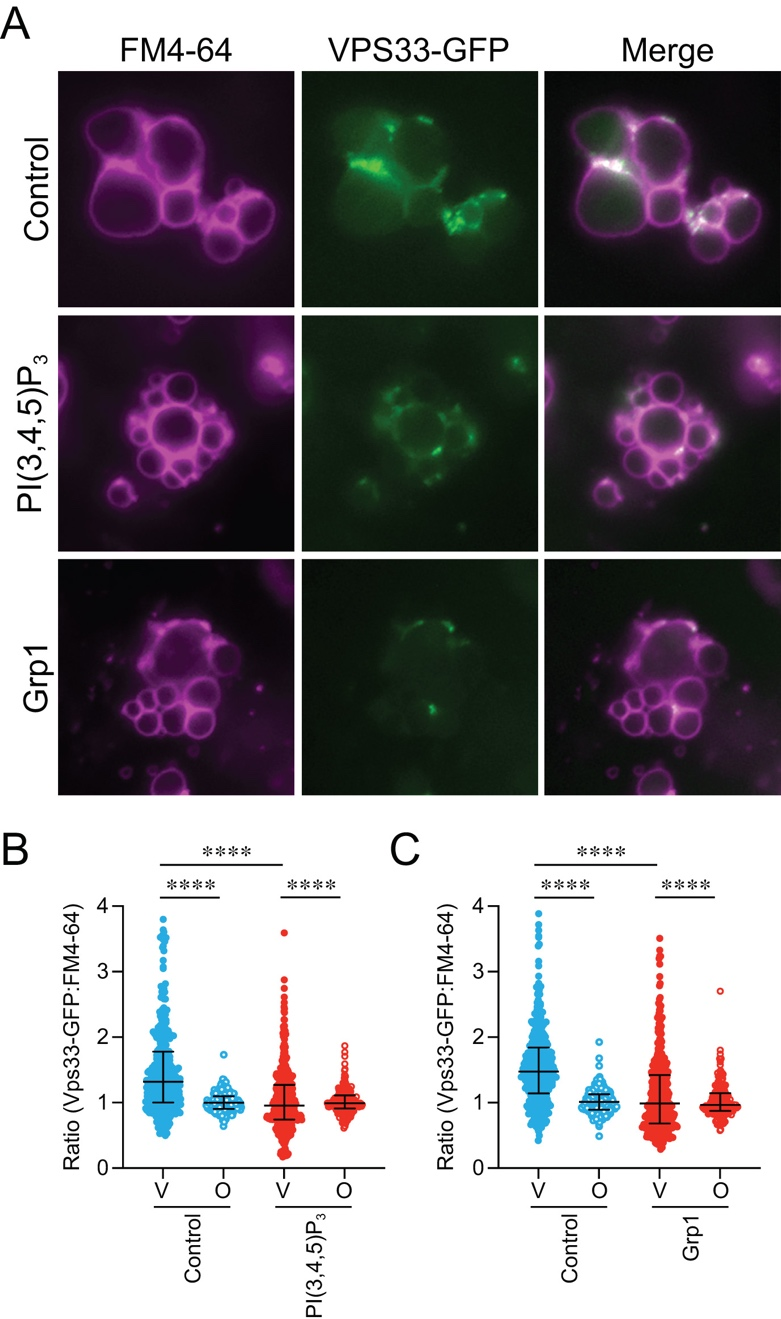
Free PI(3,4,5)P_3_ is required for Vps33 localization to vertex microdomains. **(A)** Docking reactions were performed with vacuoles harboring Vps33-GFP in the presence buffer (control), 250 μM C8-PI(3,4,5)P3 or 2 μM Grp1-PH At the end of the incubation period, reactions were processed for fluorescence microscopy as described above. **(B-C)** Quantitation of ratiometric fluorescence intensities of vertices (V) and outer edge (O) in panel (A). Error bars represent geometric means ± geometric SD (n=3). Significance was determined using one-way ANOVA for multiple comparisons and Tukey’s post-hoc test for individual *p*-values. **** *p* < 0.0001; Scale bars: 5 μm.

While examining vacuole docking with inhibitory levels of PTEN, Grp1-PH and C8-PI(3,4,5)P3 we observed a reduction in the vacuole clusters as well the number of vacuoles per cluster. This suggested that PI(3,4,5)P_3_ could affect docking/tethering. For this we used vacuoles harboring GFP-Ypt7 and examined its enrichment at vertices when PI(3,4,5)P_3_ was sequestered by 2 μM Grp1-PH. This showed that sequestering PI(3,4,5)P_3_ with Grp1-PH blocked GFP-Ypt7 from becoming enriched at vertex domains (Fig. 6A–B). GFP-Ypt7 was still visible on Grp1-PH treated vacuoles; however, the intensities at vertex sites were diminished while intensities at the outer edges were increased. To confirm that endogenous PI(3,4,5)P_3_ availability affected GFP-Ypt7 distribution we used 250 μM C8-PI(3,4,5)P_3_ as a competitive inhibitor. This showed that the presence of C8-PI(3,4,5)P_3_ inhibited the vertex enrichment of GFP-Ypt7 at vertex domains (Fig. 6C–D). GFP-Ypt7 was distributed throughout the membranes of docked vacuoles. This was like the effects of Grp1-PH, suggesting that free PI(3,4,5)P_3_ was needed for the assembly of a fully functioning vertex microdomain.

To see if other vertex components were affected by PI(3,4,5)P_3_ we used vacuoles that contained Vps33-GFP, a HOPS subunit. Vps33-GFP localization to vertices has been shown to be sensitive to the lipid binding probes FYVE, ENTH, C1b and filipin that bind PI3P, PI(4,5)P_2_, DAG and ergosterol, respectively (Fratti *et al*, 2004). Moreover, Vps33 can be released from vacuoles when PI3P, PI4P and PI(4,5)P_2_ are sequestered (Stroupe *et al*, 2006). Here we tested the effects of C8-PI(3,4,5)P_3_ and Grp1-PH on Vps33-GFP distribution. Untreated vacuoles contained enriched Vps33 at vertex sites as previously reported (Fig. 6A–B). However, Vps33-GFP vertex enrichment was sharply reduced by both C8-PI(3,4,5)P_3_ and Grp1-PH, indicating that free native PI(3,4,5)P_3_ was required for normal vertex assembly.

### PI(3,4,5)P_3_ affected trans-SNARE pairing.

**Figure 7.**
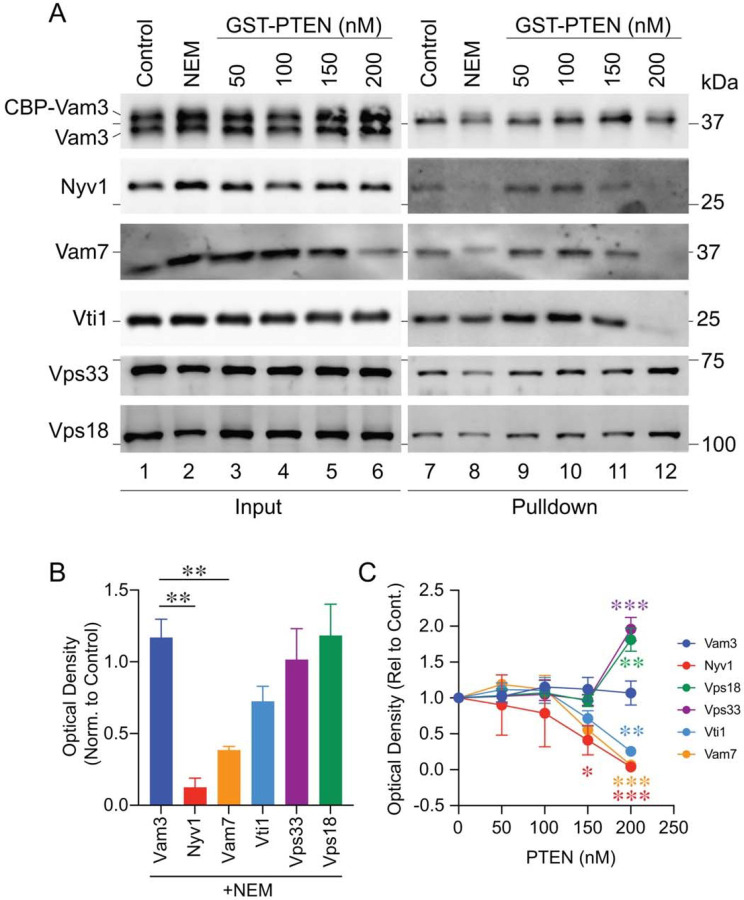
PTEN inhibits trans-SNARE pairing. **(A)** Fusion reactions were treated with buffer alone (Control), 2 mM NEM, or GST-PTEN at the indicated concentrations. Reactions were incubated for 60 min at 27°C. Protein complexes containing CBP-Vam3 were isolated with calmodulin beads. Proteins were resolved by SDS-PAGE, transferred to nitrocellulose and immunoblotted for Vam3, Nyv1, Vam7, Vti1, Vps33 and Vps18. **(B)** Quantitation of proteins bound to CBP-Vam3 in the presence of NEM. Data represents mean and SEM (n=3). Significance was determined using one-way ANOVA for multiple comparisons and Dunnett’s post-hoc of multiple comparison was used for individual *p*-values. ** *p* < 0.01. **(C)** Quantitation of proteins bound to CBP-Vam3 in the presence of PTEN. Data represents mean and SEM (n=3). Significance was determined using one-way ANOVA for multiple comparisons and Šidák’s post-hoc test for pairwise comparisons between CBP-Vam3 and specified proteins in the presence of PTEN for individual *p*-values. * *p* < 0.05, ** *p* < 0.01, *** *p* < 0.001.

During the docking stage of vacuole fusion, key proteins and lipids become enriched at vertex microdomains. The assembly of these domains promotes the optimal formation of trans-SNARE complexes between membranes (Collins & Wickner, 2007; Sasser *et al*, 2012). While PI(3,4,5)P_3_ played a role in the docking stage the gain of resistance experiments indicated that it continued to be important a later stage such as trans-SNARE pairing. To examine trans-SNARE pairing we used two types of vacuoles. One type lacked the R-SNARE Nyv1 and expressed Vam3 with an internal calmodulin binding peptide between the N-terminal helical H_abc_ domain and the SNARE domain (CBP-*VAM3 nyv1*Δ). The second type contained Nyv1 and unmodified Vam3 (*VAM3 NVY1*) (Collins & Wickner, 2007; Jun & Wickner, 2007). The formation of trans-SNARE complexes was detected when Nyv1 co-isolated with CBP-Vam3 bound to calmodulin beads. We found that under control conditions CBP-Vam3 indeed paired with Nyv1 from partner membranes as well as the HOPS subunits Vps33 and Vps18 (Fig. 7A & C). As a negative control we used NEM, which inhibited SNARE priming and thus prevented downstream trans-SNARE interactions. As expected, NEM treatment abolished Nyv1 co-isolation with CBP-Vam3 but had no effect on Vam3-HOPS interactions (Fig. 7A **lane 8 vs 9**, and B). This was consistent with a report showing that HOPS binds to the H_abc_ N-terminal domain of Vam3 (Lürick *et al*, 2015). Interestingly, Vam7 was depleted in Q-SNARE complexes when NEM was administered. The difference could be linked to a pool of free Vam7 bound to lipids and not SNAREs (Thorngren *et al*, 2004). This could also be due to the effect of NEM on the free cystine in Vam7 where alkylation might prevent full Q-SNARE complex formation. However, this is unlikely as the alkylated Vam7 was seen by the mobility shift of the band present in the pulldown.

To test the role of PI(3,4,5)P_3_ in trans-SNARE pairing we used a concentration curve of PTEN. This showed that increasing amounts of PTEN blocked CBP-Vam3 pairing with Nvy1, thus demonstrating that trans-SNARE complex formation was inhibited by eliminating PI(3,4,5)P_3_. This could be due to two factors that may be linked. First, the Qb-SNARE Vti1 was absent from the CBP-Vam3 complex when PTEN was present at 200 nM even though it was fully present in the input (Fig. 7A, **lane 6 vs 12**). Second, Vam7 was significantly depleted in the input membrane fraction and completely absent from CBP-Vam3 complexes when vacuoles were treated with 200 nM PTEN. It is important to note that the first step in trans-SNARE isolation is pelleting the membranes which separates vacuole-bound from unbound proteins. Thus, a release of Vam7 would result in its absence from the input blot. This was not due to protease activity as both vacuole populations lacked *PEP4*. Together, this suggested that Vam7 was released from membranes when PI(3,4,5)P_3_ was eliminated. Vam7 has been shown by others to bind PI(3,4,5)P_3_ in lipid overlay assays (Yu & Lemmon, 2001). Consequently, it could be that Vam7 bound to PI(3,4,5)P_3_ was needed for Vti1 to bind Vam3 in a 3Q-SNARE complex. We must note that Vti1-Vam3 binding was not affected by eliminating or blocking other PIs including PI3P, PI4P, and PI(4,5)P_2_ (Collins & Wickner, 2007).

In addition to SNARE complex formation CBP-Vam3 also pulled down the HOPS complex; however, when vacuoles were treated with PTEN we found that it did not inhibit HOPS co-isolating with CBP-Vam3. On the contrary, we found that depleting PI(3,4,5)P_3_ enhanced HOPS binding to CBP-Vam3 (Fig. 7A–C). This could be due to an exchange in HOPS-Vam3 interactions for binding Nyv1.

### Grp1-PH displaced Vam7 from membranes

**Figure 8.**
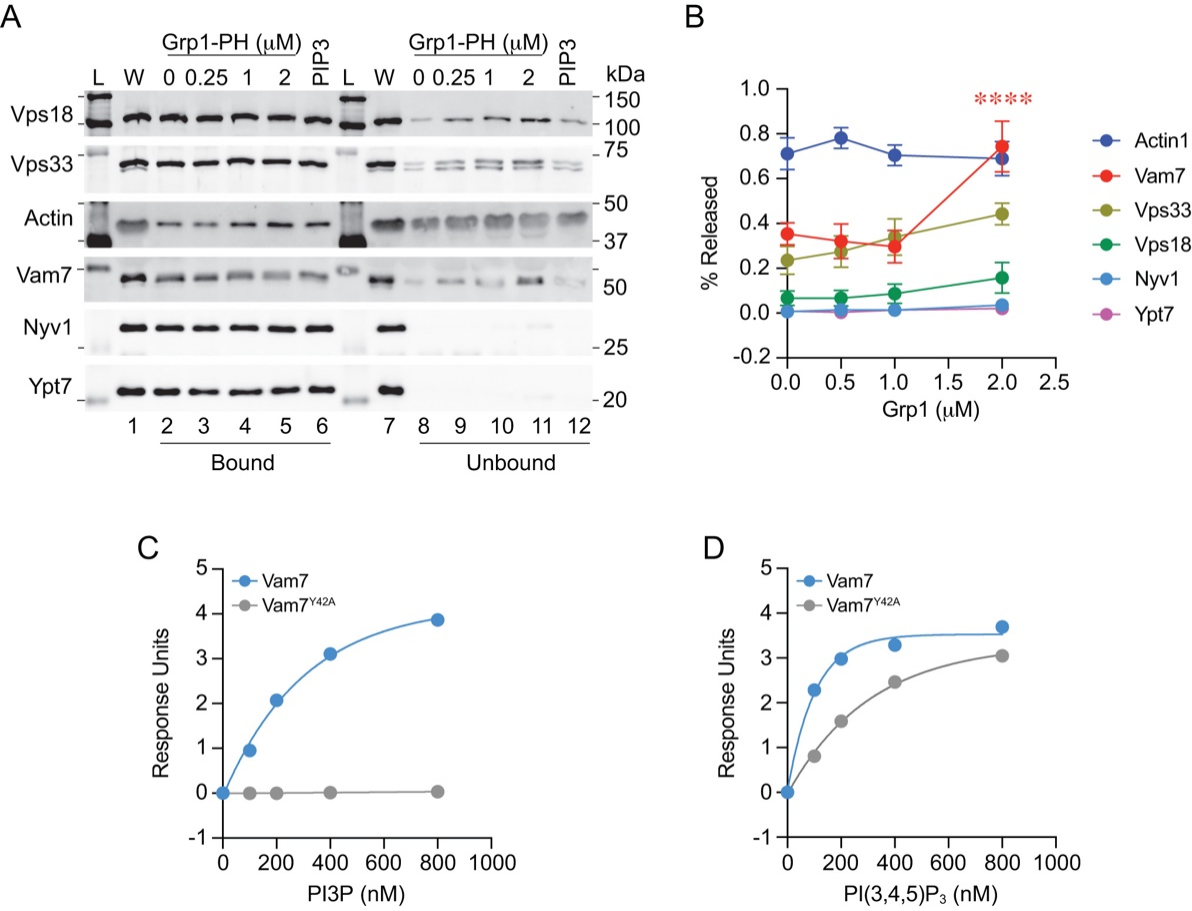
Sequestering PI(3,4,5)P_3_ with GST-Grp1-PH releases Vam7 from membranes. **(A)** Fusion reactions were treated with buffer alone (Control) or GST-Grp1-PH at the indicated concentrations. Reactions were incubated for 60 min at 27°C. Membrane-bound and unbound proteins were separated by centrifugation (16,000, 10 min, 4°C) into supernatant (unbound) and pellet (bound) fractions. Pellets were resuspended in PS buffer to match the starting volumes. Bound and unbound fractions were mixed with SDS-loading buffer, separated by SDS-PAGE, transferred to nitrocellulose and probed for with antibodies against Actin, Nyv1, Vam7, Ypt7, Vps33 and Vps18. **(B)** Quantitation of proteins released from vacuoles in the presence of Grp1-PH. Data represents mean and SEM (n=4). Significance was determined using one-way ANOVA for multiple comparisons and Šidák’s post-hoc test for pairwise comparisons between each protein released absence or presence of GST-Grp1-PH for individual *p*-values. **** *p* < 0.0001. L, ladder; W, whole reaction. **(C-D)** BLI binding curves of response units versus protein concentrations of wild type Vam7 and Vam7^Y42A^.

To verify that the loss of Vam7 from vacuoles treated with PTEN was due to eliminating PI(3,4,5)P_3_ we tested protein binding in the presence of Grp1-PH. Fusion reactions were treated with buffer alone (0 μM) or GST-Grp1-PH at increasing concentrations and incubated at 27°C for 60 min. After incubation, vacuole-bound and unbound proteins were separated by centrifugation, and the two fractions were examined by Western blotting. As seen previously, populations of soluble proteins were seen in both pellet (bound) and supernatant (unbound) fractions including Vps18 and Vps33, actin, and Vam7 (Fig. 8A–B). In contrast, the membrane-anchored proteins Nyv1 and Ypt7 were only seen in the bound fraction. Treatment with GST-Grp1 only affected Vam7 binding where significantly more was present in the supernatant and depleted from the bound fraction (Fig. 8 **lanes 5 and 11**). This was reminiscent of a previous study showing that Vam7 was released in the presence of the lipid binding domains FYVE, ENTH and C1b, which bind PI3P, PI(4,5)P_2_ and DAG, respectively (Fratti *et al*, 2004). That said, HOPS binding was not altered in the presence of Grp1-PH, whereas a previous study showed that Vps33 was released by FYVE, ENTH as well as the PI4P binding domain Fapp1-PH (Stroupe *et al*, 2006). We also tested C8-PI(3,4,5)P_3_ at 250 μM which inhibits fusion. Unlike Grp1-PH, C8-PI(3,4,5)P_3_ had no effect on Vam7 binding. This could be due to differences in binding affinities where C8-PI(3,4,5)P_3_ cannot compete with full length lipid. This also indicated that separate factor required for fusion was blocked by C8-PI(3,4,5)P_3_ such as the mislocalization of Ypt7 and Vps33. Together these data suggested that Vam7 release could be due to a direct interaction and not to a general blockage of vertex microdomain assembly/maintenance.

### Vam7 binds PI(3,4,5)P_3_

Vam7 uses its PX domain to bind the vacuole membrane through its interactions with PI3P, and mutating Tyr42 to Ala inhibits binding and fusion though not entirely (Cheever *et al*, 2001; Boeddinghaus *et al*, 2002; Fratti & Wickner, 2007). The ability of Vam7^Y42A^ to form complexes with SNAREs and HOPS was attributed to its directly binding to proteins without the aid of PI3P binding. That said, it could be that Vam7^Y42A^ interacted with other lipids, including PI(3,4,5)P_3_. Previously we showed that Vam7 also binds to PA and PI5P by liposome flotation, albeit to a lower extent versus PI3P (Miner *et al*, 2016). We also observed weak PA binding by surface plasmon resonance, microscale thermophoresis and bio-layer interferometry (BLI) (Sparks *et al*, 2019, 2022; Calderin *et al*, 2025). This suggests that Vam7 could have a second lipid binding site or promiscuous binding by a single site. The former is in accord a study showing that many PX domains have a second lipid binding site (Chandra *et al*, 2019).

Here we compared PI3P and PI(3,4,5)P_3_ binding using streptavidin coated BLI probes bound to biotinylated lipids. These were incubated with GST-Vam7 and GST-Vam7^Y42A^ at different concentrations to measure binding. Here we show curves of response units versus protein concentration. This illustrated that Vam7 strongly bound to PI3P (K_D_ ~200 nM) whereas Vam7^Y42A^ failed to bind as expected (Fig. 8C). In comparison, we found that both Vam7 and Vam7^Y42A^ bound to PI(3,4,5)P_3_ with K_D_ values of ~70 and ~210 nM, respectively (Fig. 8F). The binding of Vam7^Y42A^ to PI(3,4,5)P_3_ suggested that the PX domain could have a second binding site. Alternatively, other residues in the same binding pocket might engage the D4 and D5 phosphates of PI(3,4,5)P_3_, which are not involved in binding with the D3 phosphate of PI3P. It is also possible that the Y42A mutation weakened PI(3,4,5)P_3_ binding without abolishing it. Future investigation will explore these options.

## DISCUSSION

In this study we asked if there was a role for PI(3,4,5)P_3_ in yeast vacuole fusion. In previous studies we have shown that adding C8-PA and C8-PI(3,5)P_2_ inhibited fusion at the pre-priming stage and between SNARE pairing and hemifusion, respectively (Starr *et al*, 2016; Miner *et al*, 2019). Here we saw that C8-PI(3,4,5)P_3_ also inhibited fusion after the GDI-sensitive/Ypt7-dependent step. Separately we found that sequestering native PI(3,4,5)P_3_ with Grp1-PH or modifying it with PTEN inhibited fusion. Furthermore, blocking PI(3,4,5)P_3_ interactions with unknown endogenous binding partner(s) with PIT-1 and DMPIT rescued inhibition by Grp1 and PTEN. Together these data indicate that vacuoles have an endogenous pool of PI(3,4,5)P_3_ and that it interacts with a least one vacuolar protein to promote optimal fusion.

Stage specific experiments showed that both Ypt7-dependent tethering and trans-SNARE pairing required PI(3,4,5)P_3_. The Ypt7-dependent effect was shown by the lack of its enrichment at vertices. Docked vacuoles form vertex microdomains that become enriched in Ypt7, HOPS, SNAREs and actin, as well as PI3P, PI(4,5)P_2_, DAG, and ergosterol (Wang *et al*, 2002, 2003; Eitzen *et al*, 2002; Karunakaran *et al*, 2012; Fratti *et al*, 2004). The composition of the vertices is critical, highly regulated, and interdependent, meaning that the enrichment of key proteins and lipids will consequently affect the accumulation of other proteins and lipids at the site. The correct localization of proteins and lipids at vertex sites promotes trans-SNARE pairing and fusion. Thus, it follows that a reduction in functional vertex sites led to inhibiting trans-SNARE pairing. Interestingly though, the block in trans-SNARE pairing was accompanied by a loss of Vam7 from the membrane and the loss of Vti1 from the 3Q SNARE bundle.

### Vam7 and PI(3,4,5)P_3_

The interaction between Vam7 and PI3P has long been recognized as the mechanism by which this soluble SNARE associates with membranes prior to its interactions with its cognate SNAREs and HOPS (Cheever *et al*, 2001; Boeddinghaus *et al*, 2002). Its lipid binding capacity lies in its N-terminal PX domain and PI3P binding can be largely abolished by mutating Tyr42 to Ala. That said, Vam7^Y42A^ can still associate with vacuoles and support fusion albeit at reduced levels (Fratti & Wickner, 2007). This was attributed to its interactions with proteins and not other lipids. Here we showed that Vam7 and Vam7^Y42A^ can both bind to b-PI(3,4,5)P_3_ by BLI implying that a second binding site exits as seen with other PX domains (Chandra *et al*, 2019). An alternative explanation could lie in the use of additional residues in the same binding pocket to accommodate PI(3,4,5)P_3_ binding that are not involved in PI3P binding. Thus, mutating Tyr42 to Ala blocked PI3P binding while only attenuating PI(3,4,5)P_3_ binding.

Although Vam7 strongly bound to PI(3,4,5)P_3_ we showed that it can be released from vacuoles when native PI(3,4,5)P_3_ is blocked Grp1-PH or modified with PTEN. This poses the question of why two lipid interactions are needed? While PI3P is delivered to vacuoles via endolysosomal trafficking it is in low concentration and a second round of PI3P is produced on site (Thorngren *et al*, 2004). It is possible that the ebb and flow of PI3P levels could negatively affect Vam7 recruitment to vacuoles and that a second ligand is needed for stable association with the membrane. Originally, we hypothesized that PA could be the initial binding partner and that its conversion to DAG by Pah1 would lead to a hand off to PI3P. This was based on the required conversion of PA to DAG during pre-priming and the idea that Vam7 only binds one lipid at a time (Sasser *et al*, 2012; Starr *et al*, 2019, 2016). Now we must consider that PI(3,4,5)P_3_ could be the binding initiator, and that Vam7 could bind two lipids simultaneously.

Another question to consider is whether Vam7-PI(3,4,5)P_3_ interactions affect Ypt7 localization. It could be that changes in Vam7 conformation induced by PI(3,4,5)P_3_ binding influences how HOPS interacts with Ypt7. This is not unlikely as both Vam7 and Ytp7 interact with the HOPS complex although not necessarily at the same time as seen by pulldown experiments (Price *et al*, 2000a; Seals *et al*, 2000; Brett *et al*, 2008; Stroupe *et al*, 2006; Collins *et al*, 2005; Fratti & Wickner, 2007; Fratti *et al*, 2007). Thus, blocking PI(3,4,5)P_3_-Vam7 binding could prevent HOPS and Ypt7 retention at vertex sites. A more direct effect would through disrupting direct Vam7-Ypt7 binding. A yeast-two-hybrid screen has shown that Vam7 and Ypt7 interact directly (Uetz *et al*, 2000). This scenario is less likely as the screen did not include PI(3,4,5)P_3_ binding. Furthermore, Vam7 pulldowns have failed to show Ypt7 (Stroupe *et al*, 2006).

Finally, how does PI(3,4,5)P_3_ affect 3Q-SNARE complex formation? Our data showed that PTEN led to the exclusion of Vti1 and Vam7 from CBP-Vam3 complexes. The absence of Vam7 is attributed to its release from the membrane, but Vti1 has a transmembrane domain (TMD) and was not displaced from vacuoles. Interestingly, the hydrophilic helical region adjacent to the Vti1 TMD has a poly basic region (PBR) with 5 Lys and Arg that could participate in binding negatively charged lipids including PI(3,4,5)P_3_, however this remains to be tested. Other SNAREs including Syntaxin-1 and Syntaxin-17 have TMD-adjacent PBRs that bind to PIPs to promote function in chromaffin granule secretion and autophagy, respectively (Laczkó-Dobos *et al*, 2024; Lam *et al*, 2008). Future studies will test whether the Vti1 PBR-membrane interaction promotes binding to Vam3 followed by Vam7, if Vam7 binds Vam3, or if complex formation is concurrent.

In conclusion, this study has shown that PI(3,4,5)P_3_ is critical in the regulation of vacuole homotypic fusion at multiple steps. While this study ends at the trans-SNARE complex stage, the gain of resistance data suggest that additional down-stream stages could be affected by PI(3,4,5)P_3_. Beyond vacuole fusion it is likely that PI(3,4,5)P_3_ signals through the yeast homologs of PDK1 (Pkh1/2), AKT1 (Sch9), PKC (Pkc1) and MAPK (Fus1, Kss1, Mpk1) to influence other functions such as autophagy, Ca^2+^ transport and actin remodeling (Brady *et al*, 2006; Inagaki *et al*, 1999; Ni *et al*, 2013; Levina *et al*, 2022; Asano *et al*, 2008; Dieterle *et al*, 2014; Kassouf *et al*, 2015; Hsu *et al*, 2000).

## MATERIALS AND METHODS

### Reagents.

Reagents were solubilized in PIPES-Sorbitol (PS) buffer (20 mM PIPES-KOH, pH 6.8, 200 mM sorbitol) with 125 mM KCl unless indicated otherwise. PIPES [Piperazine-N-N’-bis(2-ethanesulfonic acid)], HEPES [N-(2-Hydroxyethyl)piperazine-N′-(2-ethanesulfonic acid)], NEM (N-ethylmaleimide), Coenzyme A (CoA), Creatine kinase, and reduced glutathione were purchased from Sigma (St. Louis, MO) and dissolved in PS buffer or DMSO. Sorbitol, ATP, Yeast extract, Tryptone, Glucose Tris base, Triton X100 and DTT were purchased from RPI (Mount Prospect, IL). FM4–64, Goat anti-rabbit IgG (H+L) secondary antibody DyLight 650 conjugate, Goat anti-mouse IgG (H+L) secondary antibody DyLight 650 conjugate and glutathione agarose were from Thermo-Fisher (Waltham, MA). Fluorescent CF488 goat-anti GST was from Biotium (Fremont, CA). Creatine phosphate was from Abcam (Waltham, MA). PIT-1, 3,5-dimethyl PIT-1, and SAR405 were from Cayman Chemical and dissolved in DMSO (Ann Arbor, MI). C8-PC (1,2-dioctanoyl-phosphatidylcholine), C8-PE (1,2-dioctanoyl-phosphatidylethanolamine), C8-PS (1,2-dioctanoyl-phosphatidylserine) were from Avanti (Alabaster, AL). C8-PI3P (1,2-dioctanoyl-phosphatidylinositol 3-phosphate), C8-PI(3,5)P_2_ (1,2-dioctanoyl-phosphatidylinositol 3,5-bisphosphate), C8-PI(4,5)P_2_ (1,2-dioctanoyl-phosphatidylinositol 4,5-bisphosphate), C8-PI(3,4,5)P_3_ (1,2-dioctanoyl-phosphatidylinositol 3,4,5-trisphosphate), biotin-PI3P (b-PI3P), b-PI(3,4,5)P_3_, Inositol-1,3,4-trisphosphate (Ins(1,3,4)P_3_), Inositol-1,3,5-trisphosphate (Ins(1,3,5)P_3_), and Inositol-1,3,4,5-tetraphosphate (Ins(1,3,4,5)P_4_) were from Echelon (Salt Lake City UT). *p*-nitrophenyl phosphate was from MP Biomedicals (Santa Ana, Ca). Calmodulin agarose was Agilent (Santa Clara, CA). Octet Streptavidin (SA) biosensors were from Sartorius (Göttingen, Germany). Nitrocellulose was from BioRad (Hercules, CA).

### Plasmids and Recombinant proteins

Recombinant GST-Vam7, GST-Vam7^Y42A^, GDI and Pbi2 (Inhibitor of proteinase B) were prepared as described previously (Fratti *et al*, 2007; Fratti & Wickner, 2007; Starai *et al*, 2007; Slusarewicz *et al*, 1997; Miner *et al*, 2016). Plasmids to produce GST-Grp1-PH (Kavran *et al*, 1998), GST-PTEN WT, were from Addgene (Watertown, MA). The plasmid to make GST-Grp1-PH^K273A^ was a gift from Dr. N. Leslie (Lindsay *et al*, 2006). Plasmids were transformed into *E. coli* BL21 DE3 pLysS (New England Biolabs) and induced with 0.1 μM IPTG for 16h at 18°C. GST-tagged proteins were isolated using standard methods with glutathione agarose, eluted with reduced glutathione and dialyzed against PS buffer with 125 mM KCl.

### Strains and Vacuole isolation and fusion

Yeast strains (Tabel 1) were grown in YPD (1% yeast extract, 2% peptone, 2% dextrorse) or synthetic drop-out media without Trp. The pH of drop out media was adjusted to 6.0. Vacuoles were isolated as described (Haas *et al*, 1994). *In vitro* fusion reactions (30 μl) contained 3 μg each of vacuoles from BJ3505 (*PHO8 pep4*Δ) and DKY6281 (*pho8*Δ *PEP4*) backgrounds, reaction buffer 20 mM PIPES-KOH pH 6.8, 200 mM sorbitol, 125 mM KCl, 5 mM MgCl_2_), ATP regenerating system (1 mM ATP, 0.1 mg/ml creatine kinase, 29 mM creatine phosphate), 10 μM CoA, and 283 nM Pbi2 (Protease B inhibitor). Fusion was determined by the processing of pro-Pho8 (alkaline phosphatase) from BJ3505 by the Pep4 protease from DK6281. Fusion reactions were incubated at 27°C for 90 min and Pho8 activity was measured in 250 mM Tris-HCl pH 8.5, 0.4% Triton X-100, 10 mM MgCl_2_, and 1 mM *p*-nitrophenyl phosphate. Pho8 activity was inhibited after 5 min by addition of 1 M glycine pH 11 and fusion units were measured by determining the *p*-nitrophenolate produced by detecting absorbance at 400 nm.

### Trans-SNARE complex isolation.

Trans-SNARE pairing was measured as previously described with some modifications (Collins & Wickner, 2007; Jun *et al*, 2007; Qiu & Fratti, 2010; Sasser *et al*, 2012, 2013). Large scale 15X (450 μL) fusion reactions containing 45 μg of BJ3505 vacuoles (*VAM3 NYV1*) and 45 μg of BJ3505 vacuoles that lacked *NYV1* and contained Vam3 tagged with an internal calmodulin binding peptide (CBP) between the H_abc_ and SNARE domains (*CBP-VAM3 nyv1*Δ). Reactions were treated with buffer alone or 2 mM NEM as a negative control to prevent SNARE activation. Separate reactions were treated with 50, 100, 150 or 200 nM GST-PTEN. Reactions were incubated for 60 min at 27°C then placed on ice for 5 min before centrifugation (13,000 g, 15 min, 4°C) to pellet membranes. The supernatants were carefully removed, and the pellets were carefully overlaid and resuspended with 200 μL cold solubilization buffer (SB: 20 mM Tris-Cl, pH 7.5, 150 mM NaCl, 1 mM MgCl_2_, 0.5% Nonidet P-40 alternative, 10% glycerol) with protease inhibitors (1 mM PMSF and 1 cOmplete^™^ EDTA-free protease inhibitor tablet per 10 ml SB). Reactions were brought up to 600 μL with additional SB and nutated at 4°C for 20 min. Insoluble debris was removed by centrifugation (16,000 g, 20 min, 4°C). Solubilized (580 μL) material was transferred to pre-chilled tubes and 58 μL was removed from each reaction as 10% of the total reactions. CaCl_2_ was added to each sample to a final concentration of 2 mM. Next, 50 μL of calmodulin beads equilibrated with SB. Mixtures were nutated overnight at 4°C. CBP-Vam3 complexes bound material was collected by centrifugation (1,310 rpm, 2 min, 4°C) and washed 4 times with 600 μL fresh ice-cold SB. Calmodulin bound material was eluted with 1X SDS loading buffer containing 5 mM EGTA and boiled for 5 min. Samples were resolved by SDS-PAGE, transferred to nitrocellulose and probed with antibodies against Vam3, Vam7, Nyv1, Vti1, Vps33 and Vps18. Bound primary antibodies were visualized with DyLight 650-Goat anti-rabbit IgG (H+L).

### Fluorescence microscopy and Vertex microdomain formation

Isolated vacuoles were subjected to docking assays as previously described (Fratti *et al*, 2004; Wang *et al*, 2002) with slight modifications. Reactions (30 μL) contained 6 μg of vacuoles isolated from the indicated strains in fusion reaction buffer modified for docking conditions (PS buffer, 100 mM KCl, 0.5 mM MgCl_2_, 0.33 mM ATP, 13 mM creatine phosphate, 33 μg/mL creatine kinase, 10 μM coenzyme A, and 280 nM IB_2_). Measuring the vertex enrichment of factors during tethering and docking was performed with vacuoles from cells expressing GFP fusion proteins or labeled with lipid binding probes. To track GFP-Ypt7 and Vps33 localization reactions were incubated under docking conditions as described above and stained with 4 μM FM4–64 prior to examination (Wang *et al*, 2003; Fratti *et al*, 2004). To localize the distribution of PI(3,4,5)P_3_ on vacuoles, reactions were incubated with the PI(3,4,5)P_3_ binding PH domain from Grp1. GST was then visualized with fluorescent (CF488) goat-anti-GST antibody. Briefly, reactions were treated with PS buffer, DMSO, PTEN or SAR405 for 5 min, followed by the addition of 150 nM GST-Grp1-PH for 5 min. Next, CF488-anti-GST antibody was added to each reaction and further incubated for 20 min. Following incubation at 27°C for 20 min, reactions were mixed with 20 μL of 0.6% low melt agarose in PS buffer melted at 50°C and cooled to prior to mixing with vacuoles. Next, 20 μL aliquots were mounted on pre-chilled slides and observed by fluorescence microscopy. Images were acquired using a Zeiss Axio Observer Z1 inverted microscope equipped with an X-Cite 120XL light source, Plan Apochromat 63X oil objective (NA 1.4), and an AxioCam CCD camera. Quinacrine was visualized using a 38 HE EGFP shift-free filter set and FM4–64 was visualized with a 42 HE CY 3 shift-free filter set. Exposure times were set using WT vacuoles for each fluorescence channel and scripts acquired non-specific images followed by specific reporters. This ensures that bleaching is consistent to negate it as a factor in calculating intensity ratios. Exposure times were held constant within an experiment.

Images were analyzed using ImageJ software (NIH). Vertex enrichment was determined by first measuring maximum fluorescence intensity in each channel at each contact point between membranes, *i.e*., vertex domain within a cluster. Next, fluorescence intensity was measured in each channel at outer membrane domains where vacuoles are not in contact with other membranes. The ratio of specific (*e.g.*, GFP) to non-specific (*e.g.*, FM4–64) was determined for vertices and outer membrane domains and compared for relative enrichment. Measurements for each condition were taken of 15–20 clusters to yield 100–300 vertices for each condition/strain per experiment. Data from multiple experiments are combined in column plots showing individual values as well as the geometric means and geometric standard deviation for each condition.

### Western blotting

Vacuoles were solubilized with 95°C 1–5X Laemmli buffer for 5 min. Extracts were resolved using 10% SDS-PAGE and transferred to nitrocellulose for immunoblotting. Rabbit antibodies against Actin, Nyv1, Vam3, Vps18, Vps33 and Ypt7 were prepared as described (Eitzen *et al*, 2002; Nichols *et al*, 1997; Seals *et al*, 2000; Haas *et al*, 1995). Goat anti-rabbit IgG (H+L) antibody DyLight 650 conjugate was used as a secondary antibody. Fluorescence was measured with an Azure 400.

### Bio-Layer Interferometry (BLI)

Vam7 binding to lipids was measured by BLI as described (Calderin *et al*, 2025). Biotinylated PI3P and PI(3,4,5)P3 were resuspended in PS buffer to a final stock concentration of 0.1 mM. Lipids were diluted to 500 nM with BLI running buffer (PBS with 0.002% Tween-20 (v/v) and 190 μL was added to wells in a 96-well microplate. GST-Vam7, and GST-Vam7^Y42A^ was diluted to 100, 200, 400, and 800 nM with BLI running buffer and 190 μL of each dilution, for each analyte, were loaded to corresponding wells.

### Statistical analysis

Fusion results were expressed as the mean ± SEM, mean ± 95% confidence interval (CI), or geometric mean ± SD as needed. Experimental replicates (n) are defined as the number of separate experiments. For comparison of vertex enrichment all the ratio data was log-transformed to yield near-normal distribution with comparable variances. Non-parametric analysis gave indistinguishable results. Statistical analysis was performed by unpaired two-tailed t-test or One-Way ANOVA for multiple comparisons using Prism 10 (GraphPad, San Diego, CA). Statistical significance is represented as follows: *p<0.05, ** *p*<0.01, *** *p*<0.001, **** *p*<0.0001. Tukey, Dunnett, and Šidák post hoc analysis was used for multiple comparisons and individual p-values.

## Figures and Tables

**Figure 5.**
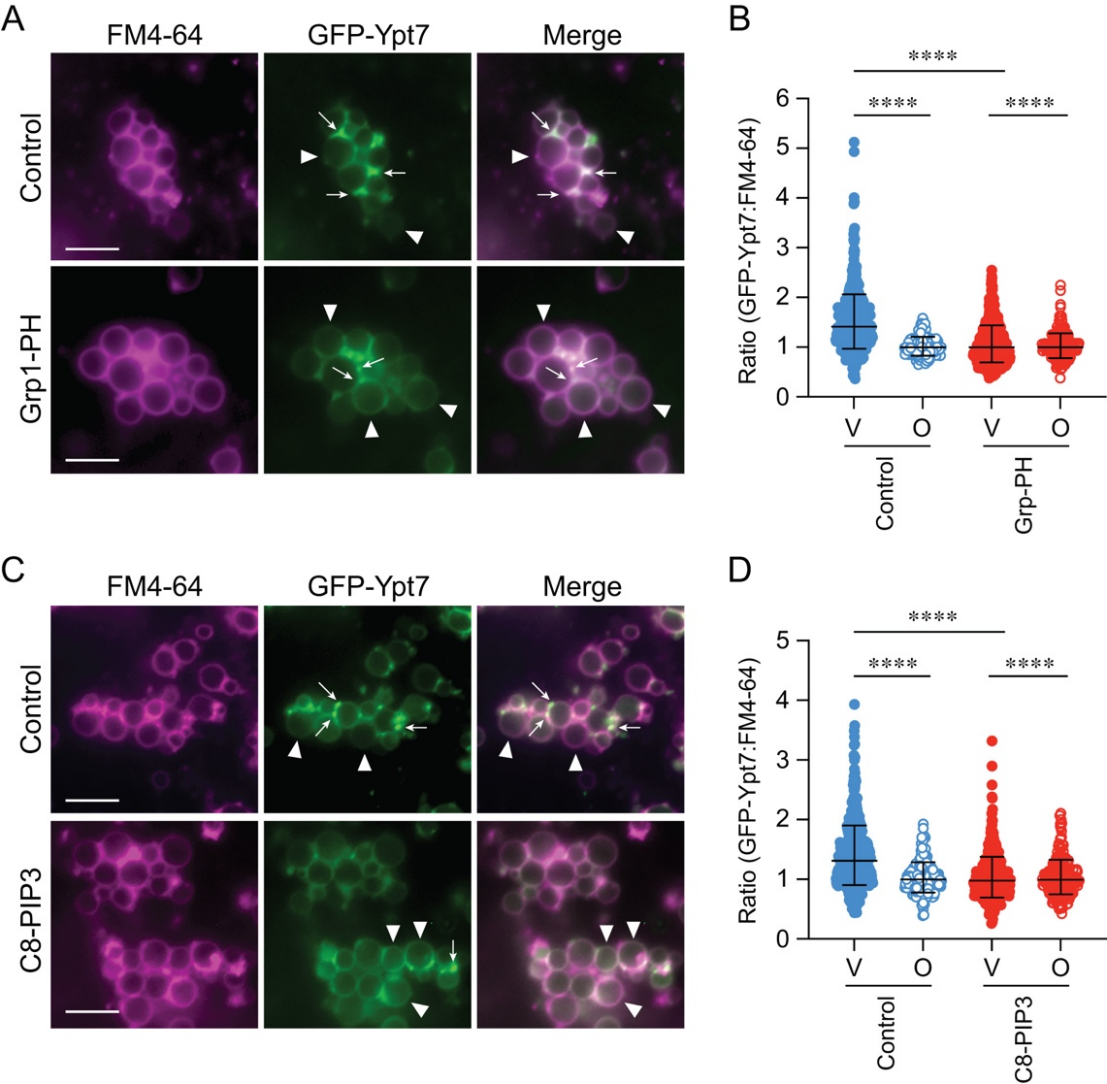
Grp1-PH blocks GFP-Ypt7 enrichment at vertices **(A)** Docking reactions with vacuoles harboring GFP-Ypt7 were treated with unlabeled 2 μM Grp1-PH to inhibit fusion or buffer and incubated for 30 min at 27°C. At the end of the incubation period, reactions were prepared for fluorescence microscopy. **(B)** Quantitation of ratiometric fluorescence intensities of vertices (V) and outer edge (O) in panel A. **(C)** Docking reactions with GFP-Ypt7 vacuoles were treated with 250 μM C8-PI(3,4,5)P_3_ to inhibit fusion or buffer and incubated for 30 min at 27°C. **(D)** Quantitation of ratiometric fluorescence intensities of vertices (V) and outer edge (O) in panel A. Data points were pooled from multiple experiments as described. Error bars represent geometric means ± geometric SD (n=3). Significance was determined using one-way ANOVA for multiple comparisons and Tukey’s post-hoc test was used for individual *p*-values. **** *p* < 0.0001. Scale bars: 5 μm.

**Table 1.** Strains used in this study.

Strain	Genotype	Source
BJ3505	*MATa pep4::HIS3 prb1*-Δ*1.6R his3-200 lys2-801 trp1*Δ*101 (gal3) ura3-52 gal2 can1*	(Jones et al., 1982)
DKY6281	*MATα leu2-3,112 ura3-52 his3*-Δ*200 trp1*-Δ*901 lys2-801 suc2*-Δ*9 pho8*Δ*::TRP1*	(Klionsky and Emr, 1989)
SEY6210	*MATα leu2-3,112 ura3-52 his3*-Δ*200 trp1*-Δ*901 lys2-801 suc2*-Δ*9*	(Klionsky and Emr, 1989)
GFP-Ypt7	*SEY6210, ypt7::HIS3 pRS304: GFP-Ypt7 (TRP1)*	(Wang et al., 2002)
Vps33-GFP	*DKY6281, VPS33-GFP*	(Wang et al., 2002)
GFP-Vps39	*SEY6210, vps39::HIS3 pYlPlac211-GFP-VPS33 (TRP)*	(Wang et al., 2002)
CBP-Vam3 *nyv1*Δ	*BJ3505, CBP-VAM3::Kan*^*r*^ *nyv1::nat*^*r*^	(Collins & Wickner, 2007)

## Data Availability

All data generated or analyzed during this study are available upon request. Addition data sharing information is not applicable to this study.

## ABBREVIATIONS

BLI: bio-layer interferometry
C8: dioctanoyl
GDI: GDP dissociation inhibitor
NEM: *N*-ethylmaleimide
NSF: NEM sensitive factor
PC: phosphatidylcholine
PE: phosphatidylethanolamine
PS: phosphatidylserine
PI: phosphatidylinositol
PTEN: phosphatase and tensin homolog deleted on chromosome 10
SNARE: soluble *N*-ethylmaleimide-sensitive factor attachment protein receptor
